# Book Reviews

**Published:** 1898-01

**Authors:** 


### Book Reviews.

HAND-BOOK OF THE MATERIA MEDICA, PHARMACY, AND THERAPEUTICS, including the Physiological Action of Drugs, the Special Therapeutics of Diseases, Official and Practical Pharmacy, and Minute Directions for Prescription Writing. By Sam'l O. Potter, A. M., M. D., M. R. C. P. Lond. Sixth edition, fully revised, and greatly enlarged. 8vo., pp. 900. Philadelphia: P. Blakiston, Son & Co. 1897. Price, $4.50.

The present edition bears very little resemblance to the first edition of ten years ago. So much has been added in the way of new material and careful revision of the old, that the edition of 1897 is practically a new work.

The information on the subject of drugs and their therapeutic application, when and how to prescribe them, is as full, complete and accessible as could be desired. The work is entirely up to date, containing the newest additions to the materia medica and will prove a valuable possession to any one who contemplates purchasing a new work on this branch.

---

A PRACTICAL TREATISE ON DISEASES OF THE SKIN. By John V. Shoemaker, M. D., LL. D. Third edition, revised and enlarged, with chromogravure plates, and other illustrations. 8vo., pp. 894. New York: D. Appleton & Co. 1897.

There is probably no work in the entire realm of dermatology which is as popular with the general practitioner as Shoemaker's. The clear clinical descriptions, often illustrated by plates, and the full and detailed therapeutic suggestions, with wealth of practical formulæ, make this work particularly to be recommended to any one who wishes to add to his library a representative work on dermatology. The present edition is an amplification of the former; some new matter and several plates have been added.

